# Content analysis of media coverage on smoke-free policy implementation in ten low- and middle-income countries

**DOI:** 10.18332/tid/211700

**Published:** 2025-11-25

**Authors:** Emily Xing, Suan Kim, Joanna E. Cohen, Tuo-Yen Tseng

**Affiliations:** 1 Institute for Global Tobacco Control, Department of Health, Behavior and Society, Johns Hopkins Bloomberg School of Public Health, Baltimore, United States

**Keywords:** secondhand smoke, LMICs, media discourse, tobacco control implementation, policy enforcement

## Abstract

**INTRODUCTION:**

Low- and middle-income countries (LMICs) face a disproportionate exposure and disease burden from secondhand smoke (SHS). Creating completely smoke-free indoor public places is effective in protecting people from SHS. This study examines how online news outlets in LMICs discuss existing smoke-free policies and their implementation.

**METHODS:**

In September 2023, we used Tobacco Watcher (www.tobaccowatcher.org), a tobacco news surveillance platform, to identify articles using the built-in topic ‘Air’ (i.e. smoke-free policy) and search terms ‘implementation’ or ‘compliance’ in 10 LMICs from 2022 to 2023. Two trained coders independently assessed all articles for their relevance (either substantially discussing in multiple paragraphs or holding a clear position on smoke-free policy implementation). A content analysis was conducted, with the coders independently coding each article for argument position, content, evaluation of current implementation efforts, and argument presenters until reaching 80% agreement or higher. Discrepancies in coding were resolved through discussion.

**RESULTS:**

Among 634 articles retrieved, 345 met the inclusion criteria. Most of these articles (81%, n=276) supported implementation of existing smoke-free policies; 31% (n=107) considered current smoke-free policy implementation efforts unsuccessful, citing lack of enforcement, signage, and other violations, while 20% (n=70) considered implementation to be successful; 21% (n=74) suggested a need for stronger smoke-free policies, including the elimination of designated smoking areas. Common argument presenters included government agencies (84%, n=291), civil society organizations (e.g. civil society: 40%, n=139), WHO (19%, n=67), and researchers or experts (18%, n=62).

**CONCLUSIONS:**

Discourse around smoke-free policy implementation in online news media of LMICs was generally supportive, praising complete bans and active implementation. However, coverage highlights that enforcement remained a challenge and pointed to a need for stronger policies. News media can be utilized as avenues for raising awareness surrounding tobacco control challenges, building support for policy, and countering tobacco industry narratives.

## INTRODUCTION

Secondhand smoke (SHS) exposure remains a major contributor to the global burden of disease, causing 1.2 million deaths every year^1^ while being associated with ischemic heart disease, stroke, lung cancer, and other serious conditions^2-4^. Low- and middle-income countries (LMICs) disproportionately bear roughly 77% of all smoking-related deaths and 89% of all SHS attributed deaths worldwide^5^. While an increasing number of countries have adopted comprehensive smoke-free policies under the WHO Framework Convention for Tobacco Control (FCTC)^6^, challenges remain in sufficient adoption and implementation of smoke-free policies in public spaces, such as lacking enforcement mechanisms, low public awareness, and tobacco industry interference^7^, especially in LMICs^8,9^.

Globally, successful implementations of smoke-free policy have resulted from strong governance, high levels of public awareness, and multisectoral collaboration from institutional and public actors to promote enforcement^10,11^. Within this framework, media can play a crucial role in shaping public discourse, communicating legislative changes, disseminating evidence, and influencing awareness and public support.

As a 2017 systematic review summarized, positive media coverage of smoke-free policies can raise salience towards SHS harms, increase public awareness, and counter tobacco use normalization^12^. Media coverage can also influence policy making; for instance, a 2010 media advocacy campaign in Mexico utilized evidence on tobacco use burden and public opinion to shift legislative vote in support for a tobacco tax, resulting in a tax increase^13^. Similarly, studies in countries like Namibia and the US have demonstrated the potential of media channels to increase pressure on governments, garner public support, counter misinformation, tailor messages to disseminate knowledge on tobacco risks, and counteract negative framing^14,15^. News outlets can serve as accessible avenues of mass promoting tobacco control efforts, with media engagement and journalist training identified as crucial strategies to garnering public support in LMICs^16^.

Several studies have investigated how tobacco control efforts are framed in the media and highlighted both opportunities and challenges associated with media portrayals. An early content analysis in Australia suggested that while tobacco control policies received overall support within media narratives, the extent of audience exposure and engagement was inconsistent^17^. Another study in the UK showed that during specific legislative events or periods of public interest, tobacco-related media coverage might increase substantially^18^. Furthermore, while advocacy groups in Australia were found to hold a notable presence in tobacco control media discourse, more research is needed to explore how different argument messengers shape media framing^19^.

In LMICs, the limited evidence available on tobacco control in the media highlights industry influence on tobacco control measures. For instance, Amalia et al.^20^ evaluated online media discourse surrounding Indonesia’s 2012 tobacco control regulation, finding divided positioning and arguments on the legislation, including tobacco industry normalization. Similarly, Robichaud et al.^21^ analyzed LMIC media coverage of the FDA’s Modified Risk Tobacco Product (MRTP) order for IQOS and found widespread misreporting. News outlets framed the FDA’s ‘reduced exposure’ order as a ‘reduced risk’ claim, reflecting media misinterpretation and industry efforts to undermine regulatory efforts. These findings reflect a need for stronger media capacity and critical reporting and mirror calls for more evidence-based narratives supporting tobacco control.

While previous studies have contributed to our understanding of media narratives in tobacco control, research remains limited in examining diverse global legislation and topics, particularly for smoke-free policies in LMICs. Research in highincome countries found that smoke-free policies were largely framed as either beneficial to public health or restrictive to individual freedoms and economic interests in the media^18,22^. However, literature on this topic disproportionately focuses on high-income countries, leaving media coverage of tobacco control in LMICs – where tobacco burden is highest – largely underexplored.

Furthermore, there is little research on how media frames the implementation and enforcement of smoke-free policies beyond the initial adoption phase. A 2022 review study found that media coverage of smoke-free policies often peaks during legislative changes but fails to be sustained during the critical implementation phases^23^. And despite spikes in coverage of tobacco control topics, news narratives fail to share clear, health-centered framing, delivering fragmented messages to the public. Additionally, implementation processes present unique challenges – such as ensuring sustained compliance, countering industry interference, and addressing local enforcement barriers – which are not fully reflected or consistently captured in media narratives.

Many studies have called for additional research to better understand the topics, framing, and nature (e.g. frequency, volume) of tobacco-related media coverage^17-23^. Research suggests that strengthening public health narratives in tobacco-related media could directly counter industry interference and garner public support^24,25^. Given media’s role in shaping public perceptions of policy success, enforcement accountability, and trust in government initiatives, such research is critical for building effective communication strategies that amplify public health narratives and better inform tobacco control agendas.

The present study addressed these gaps by systematically investigating how online news media cover smoke-free policy implementation and compliance in ten LMICs. This study employed a content analysis to identify themes, framing positions, and media actors to better understand the public discourse surrounding smoke-free enforcement efforts across LMICs.

## METHODS

In September 2023, we collected online news media articles from the Tobacco Watcher (www.tobaccowatcher.org) online tobacco-media monitoring and categorizing platform. A total of 634 unique articles published between 22 March 2022 and 22 September 2023 were identified using Tobacco Watcher’s built-in subject of ‘Air’ (referring to smoke-free-related articles) in combination with search terms ‘implementation’ or ‘compliance’, and published in one of ten LMICs: Bangladesh, Brazil, China, India, Indonesia, Mexico, Pakistan, Philippines, Vietnam, and Ukraine. These ten countries were chosen because they are among the LMICs with the highest smoking burdens^1^. We also conducted a preliminary search to ensure that a sufficient number of relevant online news media articles were available from each country during the study period.

**Figure 1:**
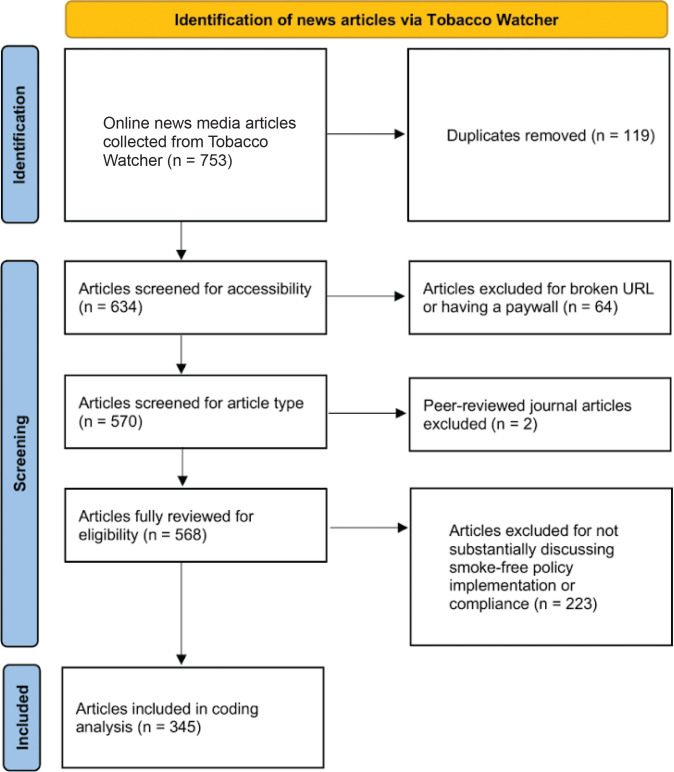
Flowchart showing the number of online news media articles on smoke-free policy implementation in ten LMICs, March 2022 to September 2023, identified at each stage of the search and screening process

Two trained coders independently assessed all articles for relevance, defined as either featuring more than one paragraph discussing smoke-free policy implementation or taking a clear position on smoke-free policy implementation (e.g. mentioning a specific policy and evaluating its impacts in fighting death/disease). Articles deemed irrelevant (n=223), inaccessible due to broken URLs (n=60) or paywalls (n=4), or identified as research or journal articles (n=2) were excluded from the analysis. This resulted in 345 of the 634 articles meeting the inclusion criteria for evaluation, as outlined in Figure 1.

Coders also classified articles by type: news article (a news report written to inform about recent events), feature article (a more creative and less formal article that explores a topic or event in greater depth than a typical news report), or opinion piece (an article written to convey an individual’s perspective on a topic). Google Translate was used to translate nonEnglish articles for content analysis.

A codebook was developed using a combination of inductive and deductive approaches through an iterative process. Codes adapted from prior research utilizing Tobacco Watcher were supplemented by variables based on themes emerged in the data through multiple rounds of preliminary coding^20,21^.

Articles were evaluated for argument positions and presenters. Variables for argument positions were: 1) supports implementation of existing smoke-free policy, promoting their continued or improved enforcement; 2) discourages implementation of existing smoke-free policy; 3) calls for changes of current policy (such as abolishing provisions allowing designated smoking areas) which suggest the need for stricter smoke-free policies to strengthen tobacco control; 4) calls for changes to current smoke-free policies by proposing a relaxation or reduction in their stringency; 5) successful implementation of smoke-free policy; and 6) unsuccessful implementation efforts for smoke-free policy. An overall argument position variable was coded as present if an article contained any of these argument positions.

Variables for argument presenters were: 1) government, including agencies at local, regional, state or country levels or officials; 2) tobacco companies or industry; 3) healthcare providers and medical professionals; 4) people who consume tobacco; 5) people who do not smoke but are affected by others’ smoking; 6) researchers/experts including professors, universities, and research institutes; 7) specific research study or research studies in general; 8) civil society organizations; and 9) the WHO. Argument presenters were coded as present if the article directly or indirectly referred to statements made by any of these presenters. Further details about each variable and examples can be found in the Supplementary file.

Articles were reviewed independently by two coders and double-coded until reaching an inter-rater reliability of ≥0.80 for all variables. Any disagreements were resolved by extensive discussion with a third coder acting as a reviewer. Coders met routinely to resolve disagreements and discuss codebook revision.

After coding 208 articles in accordance with the final iteration of the codebook, the coders reached an inter-rater reliability of 0.82–0.91 and a prevalenceadjusted kappa of 0.64–0.96 across variables, indicating fair to good agreement (see Supplementary file). The two coders then single-coded the remaining articles. Articles coded before the final codebook iteration (n=184) were recoded to ensure consistency.

Descriptive analysis was performed to obtain frequencies for each variable. Google Forms was used for data management and coding. Descriptive analysis, including frequency counts for each variable, was performed in Google Sheets using built-in functions.

## RESULTS

Among the 345 articles assessed, 94.5% were news articles (n=326), 4.3% were op-eds (n=15), and the rest were feature or other article types (n=4) (Table 1); 35.1% (n=121) of the articles were published on Indonesian platforms, 13.6% (n=47) were published in China, 7.2% (n=25) in Vietnam, 13.6% (n=47) in Mexico, 7.8% (n=27) in India, and 7.5% (n=26) in Bangladesh, with the remaining being distributed across Ukraine (9.0%, n=31), Philippines (2.9%, n=10), Pakistan (2.6%, n=9), and Brazil (0.6%, n=2). Most articles supported the implementation of smoke-free policies. Some included statements calling for stricter smoke-free policy and/or mentioned unsuccessful implementation of current policy. Some articles praised current smoke-free policy implementation, and a small portion of articles rejected current smoke-free policy measures and/or suggested relaxing these policies. We observed a variety of authorities presenting arguments, including government officials, WHO and other organizations, researchers, and health experts.

**Table 1:** Distribution of online news media articles discussing smoke-free policy implementation or compliance, by article type and country, from ten LMICs, March 2022 to September 2023 (N=345)

	*Number of* *articles*	%
**Article types**		
News	326	94.5
Op-Eds	15	4.3
Feature and other	4	1.2
**Country**		
Bangladesh	26	7.5
Brazil	2	0.6
China	47	13.6
India	27	7.8
Indonesia	121	35.1
Mexico	47	13.6
Pakistan	9	2.6
Philippines	10	2.9
Vietnam	25	7.2
Ukraine	31	9.0

Of the 345 articles included in this study, most articles (81%, n=278) contained statements supporting the implementation of smoke-free policies. Examples of these statements included those praising government programs that raised awareness for tobacco control policies, promoting collaboration across sectors for tobacco control efforts, and covering awards for successful smoke-free policy implementation. Table 2 shows example quotes for each argument position and presenter.

**Table 2:** Argument positions discussing smoke-free policy implementation found in online news media articles from ten LMICs, March 2022 to September 2023 (N=345)

*Argument position*	*Number of* *articles*	%	*Main findings*	*Example quotes*
**Support smoke-free policy implementation**	276	80.6	Argue for better implementation of current laws, including 100% smoke-free spaces	Bangladesh: ‘Speakers in the seminar presented various recommendations including strict enforcement of smoking and tobacco products control laws, encouraging tobacco farmers to cultivate alternative profitable crops, increasing anti-smoking campaigns in educational institutions, canceling the reservation of areas for smoking in public places.’
			Emphasize public health and youth protection	Indonesia: ‘In addition, the implementation of Smoke-Free Areas (KTR) as a form of respect for the right to freedom of clean air needs attention. The implementation of KTR according to the law in public places, workplaces, places of worship, children’s playgrounds, public transportation, the environment around teaching and learning places and health facilities aims to protect from exposure to other people’s cigarette smoke.’
			Call for collaboration between government, public, and other actors in creating action	Vietnam: ‘In order to contribute to protecting people from secondhand smoke and promoting smoke-free models, the Bac Ninh Center for Disease Control (CDC Bac Ninh) has coordinated with HealthBridge Canada to deploy a smoke-free tourist destination model.’
**Discourage smoke-free policy implementation**	24	7.0	Criticize smoke-free legislation as ineffective or unfair, taking away rights of both people who smoke and do not smoke	Indonesia: ‘Regional governments (Pemda) are asked to be fairer in regulating Smoke-Free Areas (KTR). Regional governments are expected to accommodate consumer interests by providing adequate smoking areas. On that basis, in its implementation, regional governments are asked to accommodate all stakeholders in the tobacco industry (IHT), including farmers and workers. “In my opinion, in order to cover these two, provide a place to reduce the losses of passive smokers. Create a smoking area, so that smokers do not use public places”.’
			Claim that bans harm businesses and the economy	Mexico: ‘I repeat, to thousands of merchants who, if they sell cigarettes, will no longer be able to display them, will no longer receive support from the brands that sponsored them, because it is already prohibited, and as for going to clubs, yes, you can, but if you had the habit of drinking and smoking on a terrace or balcony, they will not sell to you either.’
**Advocate for strengthened policies**	74	21.4	Push for stronger national and local regulations	Mexico: ‘We want to influence the legislative process and achieve the reform of article 27 of the General Law for Tobacco Control in order to establish 100% smoke-free spaces. For this reason, today we are presenting the online campaign that aims to have a constant presence to attract attention during the rest of the current legislature.’
			Call for elimination of designated smoking areas (DSAs)	Bangladesh: ‘The country’s anti-tobacco organizations have already placed six proposals on the amendment of tobacco control law including – ensuring 100 percent smoke-free environment by prohibiting smoking in all forms in public places, workplaces, and public transport; abolishing “Designated Smoking Area (DSA)”.’
			Messengers or argument presenters include government officials, health organizations, civil society, among other actors	Philippines: ‘Now is an opportune time to pass House Bill 5315 …We call on our leaders to pass this legislation and protect the policymaking process from any interference from the tobacco industry’, said Dr. Ma. Victoria Raquiza, co-convenor of Social Watch Philippines.
			Call for policy expansion to additional tobacco products, such as e-cigarettes and hookah	Philippines: ‘The conference also aims to teach the importance and the process of preparing an effective smoke-free enforcement mechanism and identify and respond to challenges in implementing policies and sustaining the smoke-free campaign with the emergence of electronic nicotine delivery systems (ENDS) and heated tobacco products (HTPs).’
**Advocate for more lenient policies**	9	2.6	Report statements discouraging policy implementation, with concerns cited about economic losses	Bangladesh: ‘Tobacco companies and their beneficiary groups are trying to mislead the public and policymakers by misrepresenting various clauses of the draft amendment through press conferences, policy dialogues, and media campaigns.’ Indonesia: ‘A number of business actors in the tobacco sector complain about this rule because it is considered to disturb the tobacco ecosystem.’
			Express concern for maintaining market fairness and personal freedoms in policy enforcement	Mexico: ‘The measure does not only impose barriers to free competition and free access, but the way in which the judges are ruling is distorting the market even more’, he says. “The establishment that obtains a suspension has an advantage over its neighbor, because there are cases in which the establishments are next to each other, but only one can offer food and beverage service with areas for smokers”, explains the lawyer, who handles the legal proceedings of restaurants, casinos, and hotel chains.’
**Successful implementation of smoke-free policy**	70	20.3	Highlight impact of strict smoke-free laws and enforcement efforts on increased compliance and smoking reduction	Bangladesh: ‘With acts like The Smoking and Using of Tobacco Products (Control) Act, 2005 and The Juvenile Smoking Act, 1919, Bangladesh has successfully reduced smoking from a reported 34 percent to 20 percent within the span of 13 years (2000–2013).’
			Recognition of tobacco control successes by credible public health organizations (e.g. WHO, PAHO) highlights best global practices	Mexico: ‘The Pan American Health Organization (PAHO) has issued a statement welcoming Mexico’s tightening of its smoking ban.’ China: ‘The Shanghai Regulations on Smoking Control in Public Places came into effect on 1 March 2010. It is the first local regulation on smoking control promulgated by the provincial people’s congress in mainland China after the WHO Framework Convention on Tobacco Control came into effect in my country. The revised Regulations came into effect on 1 March 2017, achieving a comprehensive indoor smoking ban. The World Health Organization awarded the Shanghai Municipal People’s Government the World No Tobacco Day Award.’
			Educational and grassroots campaigns effectively build public support for smoke-free policies	Ukraine: ‘On June 23, a round table “Strengthening the protection of the population of Kyiv against the effects of secondary tobacco emissions and electronic smoking devices” was held in the premises of Vcentri HUB (KNP Communication Center), which was initiated by the all-Ukrainian campaign “Youth free from smoking” in partnership with the NGO Civil Center representative office “Life”. The participants of the event agreed on steps to intensify cooperation in the field of informing the population of Kyiv about the requirements of the antitobacco legislation, in particular, placing informational notices about the ban on smoking at public transport stops and educational work with legal entities to place notices in their enterprises and institutions.’
**Unsuccessful implementation of smoke-free policy**	107	31.0	Current tobacco control laws contain loopholes that tobacco industry exploits	Bangladesh: ‘Tobacco companies are taking advantage of loopholes in existing tobacco control laws. As a result, the purpose of the legislation is not being achieved or the benefits expected. Achieving the desired goals of tobacco control requires coordination and the removal of barriers. One way around the hurdle is to amend existing tobacco control laws to make them stronger as soon as possible.’
			Weak enforcement leads to continued smoking violations, with compliance issues in DSAs, schools, and other public places	China: ‘On 30 April, the Beijing Tobacco Control Association released the latest tobacco control complaint data: from 1 March to 30 April, a total of 1983 complaints were received, an increase of 16.9% month-on-month. Among them, illegal smoking in public places increased significantly month-on-month. First, the total number of complaints and reports on violations of Beijing’s tobacco control regulations was 1983, up 16.9% month-on-month. Second, complaints and reports on illegal smoking in public places such as restaurants, offices, and office buildings increased by 14.9%, 44.6%, and 28.1% month-on-month, respectively.’
			Smoke-free policies are further hindered by lack of public awareness and cultural norms regarding tobacco use	Ukraine: ‘Natalia from Lvov shared with us that, as a regular customer of one of the cafes, she is openly allowed to smoke: “They allocate tables in the far corner for this. I have lunch there almost every day, and they don’t want to lose a customer, so they take the risk”. One of the restaurateurs told us that he allegedly rented out part of his restaurant to another company, and people smoke in that part. “And if the inspectors bring charges, then let them look for the owner of that company”.’

Arguments that were frequently presented as reasons for supporting smoke-free policy included protecting youth, eliminating tobacco-related death and disease, protecting tourism interests, upholding the right to breathe smoke-free air, and protecting the environment.

A substantial number of articles included statements calling for stricter smoke-free policy (21.4%, n=74) and statements mentioning unsuccessful implementation of current policy (31.0%, n=107). These articles sometimes argued that despite tobacco policies being sufficiently strict on paper, implementation efforts were weak (‘paper tiger’). Furthermore, many articles covered attempts of tobacco companies and other businesses to take advantage of legislative loopholes. We observed a diverse set of article types and argument presenters (government officials, researchers, civil society organizations such as NGOs, etc.) calling for stricter smoke-free policy.

Discussion attributed suboptimal implementation of law to lack of public awareness, lack of government authority or incentive to enforce legislation, and prevailing cultural norms that result in violations.

We observed articles discussing 100% smoke-free spaces and the abolition of designated smoking areas (DSAs), drawing on scientific evidence and personal anecdotes. Some articles called for a national smoke-free law to supersede regional interests, citing regional disparities in tobacco control implementation. Furthermore, we observed calls to extend current smoke-free policies to additional forms of tobacco.

There were also articles praising existing smoke-free policy implementation, stating it is being carried out well (20.3%, n=70). These articles presented scientific evidence of a decrease in smoking prevalence, awards from organizations or governments (e.g. WHO, Health Ministry), successful events or campaigns, and new tools, mostly seen in stories at a local level.

A small portion of articles rejected current/existing smoke-free policy measures in general (7%, n=24) and suggested altering current smoke-free policies by relaxing their stringency (2.6%, n=9). These articles cited businesses or tobacco industry actors that resisted stringent laws due to economic interests. Other arguments against stronger policies included bribery, and corruption, and taking away the rights of people who smoke.

A wide variety of authorities and entities were used to present arguments. Government agencies, officials, and affiliated organizations were most frequent argument presenters (84.3%, n=291), followed by civil society organizations (36.2%, n=125) and WHO (20.3%, n=80), and then by researchers/experts (18.0%, n=62) and research/studies (15.1%, n=52). Doctors/hospitals (6.7%, n=23) and people who consume tobacco (3.8%, n=13) were also mentioned. The tobacco industry was least cited (1.7%, n=6). Table 3 presents examples in which each argument presenter was used to support claims.

**Table 3:** Argument presenters discussing smoke-free policy implementation, found in online news media articles from ten LMICs, March 2022 to September 2023 (N=345)

*Argument* *presenters*	*Number of* *articles*	%	*Main findings*	*Example quotes*
**Government**	291	84.3	Governments generally support strict tobacco control laws to combat secondhand smoke (SHS) exposure	Vietnam: ‘From 1 August, the Ministry of Health will ban smoking in all public areas, schools, medical and healthcare facilities, children’s playgrounds and centers, flammable and explosive areas, work offices and buildings, and public vehicles.’
			Discuss ongoing enforcement measures by government, e.g. authority inspections for compliance, awareness campaigns, and expert workshops, highlighting gaps and successes	Mexico: ‘He said that inspectors from the State Commission for the Protection against Sanitary Risks (Coepris) regularly visit restaurants to monitor compliance with the Law, without any anomalies having been reported so far.’
			Highlight awards or recognition received by governments for successful implementation efforts, such as the ASEAN Smoke-Free Awards or WHO WNTD Award	Indonesia: ‘The Mimika Regency Government received an award from the Ministry of Health for its efforts to implement a smoke-free area.’
**Civil society organizations**	125	36.2	NGOs and civil society organizations are strong advocates of smoke-free policies, emphasizing the value of protecting public health	Ukraine: ‘Public organization “Life” held a press conference on the restoration of control over compliance with the smoking ban in the premises of restaurant establishments.’
			A wide range of organizations are represented, including grassroots advocacy groups for tobacco control, research-oriented NGOs, etc.	Vietnam: ‘Here, the Provincial Steering Committee for Tobacco Harm Prevention called on departments, branches, sectors and organizations to strengthen the dissemination of the Law on Tobacco Harm Prevention, recommendations of the Ministry of Health, and the harmful effects of tobacco use; strictly implement regulations on tobacco harm prevention and smoke-free environments according to the law; hang signs at places where smoking is prohibited.’	
			Reports from the community and civil society organizations highlight inconsistent enforcement and disparities in compliance with smoking bans, often calling for stricter enforcement or policy	Bangladesh: Iqbal Masud, Director of Health and Wash Sector of Dhaka Ahsaniya Mission, stated: ‘It is never possible to protect non-smokers from secondhand smoke by maintaining ‘designated smoking areas’.	
**WHO**	80	23.2	The WHO strongly supports comprehensive smoke-free policies for all public places, with the WHO FCTC being an important reference for national tobacco control goals	Mexico: ‘WHO welcomes such a bold move on tobacco control. We call on all countries to strengthen No Tobacco policies and help us prevent 8 million deaths every year.’	
			The WHO supports innovative practices for control measures and government efforts to implement smoke-free policies	Indonesia: ‘The KTR e-money dashboard is the result of collaboration between the Ministry of Health and WHO in developing an online and mobile media applicationbased instrument to monitor the performance of local governments in supervising and enforcing KTR regulations.’	
**Researchers/experts**	62	18.0	Researchers and experts forward evidence-based recommendations for the effective implementation of smoke-free laws	Bangladesh: ‘Before this, in the presentation of the main article, the important aspects of the amendment of the Tobacco Act were presented by the executive director of Health Protection Foundation and public health expert Dr. Nizam Ahmed. There are also some loopholes in the existing law. Several recommendations are also presented. Among these, things like banning smoking in public, banning the display of tobacco products in sales centers, banning the retail sale of bidi-cigarettes, banning e-cigarettes and heated tobacco are noteworthy.’	
			Serve as authority-building personnel in presenting claims and communicating policy impacts	China: ‘Our city also takes advantage of the coordinated development of Beijing, Tianjin and Hebei, and plays the role of think tanks of experts and scholars from the three regions. During the drafting of the “Measures”, we were fortunate to receive strong support from smoking control experts from the three regions and the research team of Yanshan University.’	
			Prioritize public health values, advocating for stronger tobacco control while highlighting current successes, failures, and shortcomings	Indonesia: ‘Dr. Tuti Budi Rahayu, Lecturer in Sociology, Faculty of Social and Political Sciences, Airlangga University (FISIP Unair) on Radio Suara Surabaya said that the policy actually already has a Law that regulates it. However, so far Tuti considers its implementation to be less effective and tends to be underestimated.’	
**Research/studies**	52	15.1	Provide data and scientific evidence in support of claims made in articles, such as supplementing calls for 100% smoke-free policy through highlighting research on the non-compliance of current designated smoking areas	Bangladesh: ‘A recent study conducted by PROGGA (Knowledge for Progress) and the Johns Hopkins Bloomberg School of Public Health sheds light on how designated smoking areas in hotels, restaurants, and trains can still leave non-smokers vulnerable to secondhand smoke. Out of the 526 venues visited, the study examined 41 designated smoking areas (DSAs). None of them fully complied with the required measures outlined in the tobacco control law.’	
			Studies highlight weak enforcement, public non-compliance, health risks, tobacco industry interference, and economic losses related to SHS exposure	India: ‘Our recent study has shown that the annual direct economic costs attributable to Second-Hand Smoking in India amounted to Rs 567 billion. This accounts for 8 per cent of total annual health care expenditures on top of the staggering Rs 1773.4 billion (US$27.5 billion) in annual economic burden from tobacco use.’ (Dr. Rijo M John, Rajagiri College of Social Sciences and Economist and Health Policy Analyst and Researcher)	
			Reflect public opinion, informing sentiment and awareness towards current tobacco control efforts and potential changes	Ukraine: ‘According to a representative survey conducted by the Kyiv International Institute of Sociology (KIIS) in May 2022 among residents of Ukraine (aged 18 and older), the vast majority of respondents (80%) support the [proposed] ban on smoking electrically heated tobacco products in public places (they are smoked with devices presented in Ukraine by the brands glo, IQOS).’	
**Doctors/hospitals**	23	6.7	Medical professionals emphasize the need to fully protect civil society and public health rights, proposing measures such as stricter bills	Philippines: ‘Now is an opportune time to pass House Bill 5315. Smoke-Free laws have been shown to reduce smoking initiation and exposure to secondhand smoke. We call on our leaders to pass this legislation and protect the policy-making process from any interference from the tobacco industry’, said Dr. Ma. Victoria Raquiza, coconvenor of Social Watch Philippines.	
**People who do not smoke but are affected by tobacco**	15	4.3	People who do not smoke face significant risks from SHS exposure, even with DSAs due to poor compliance and improper enforcement, violating health rights	Vietnam: ‘To prevent and combat the harmful effects of tobacco smoke and tobacco-related diseases, it is best to create a smoke-free environment. Create a clean working environment, ensure the rights of non-smokers to breathe fresh air without tobacco smoke; help reduce the rate of tobacco use, reduce the rate of illness and death from tobacco-related diseases for workers.’	
			Anecdotes from people who do not smoke, including from groups vulnerable to SHS such as mothers, are used to support tobacco control claims	Bangladesh: ‘We were having difficulty in breathing and it was causing extreme discomfort to our two children. The entire restaurant atmosphere turns very uncongenial to us like non-smokers and it caused very negative impact on our appetite’, said Nasima, a 28-year-old woman who went to a restaurant with her family.	
			People who do not smoke strongly support smoke-free environments	Indonesia: ‘We want to narrow the space for smokers because there is a right for non-smokers to breathe free air.’	
**People who consume tobacco**	13	3.8	People who consume tobacco show resistance against smoking bans and frustrations at their freedoms being limited	Mexico: ‘But some smokers are dismayed by the draconian nature of the new law. It essentially means many will only be able to smoke in their own homes or other private residences.’	
			There exist persistent non-compliance and disregard for smoking regulations	China: ‘However, four years later, everyone seems to have forgotten the ban. The no-smoking signs in restaurants are still there, but smokers turn a blind eye and continue to puff away smoke as usual.’	
**Tobacco industry**	6	1.7	Tobacco companies are actively resisting complete smoking bans and exploiting current policy loopholes, hindering effective implementation	Bangladesh: ‘Tobacco companies are taking advantage of loopholes in existing tobacco control laws. As a result, the purpose of the legislation is not being achieved or the benefits expected.’	
			Argue that strict smoke-free measures hurt businesses financially, voicing concerns regarding market unfairness, revenue losses etc., to the public	Mexico: ‘Some are fighting the ban, citing potential business losses. Oxxo, a large chain of 20000 convenience stores throughout Mexico, is asking to be exempt from the regulations.’	

## DISCUSSION

In this study, discourse around smoke-free policy implementation in online news media of LMICs has been generally supportive, praising active implementation efforts and complete bans. New smoke-free legislation, awards received for positive implementation, insufficient policy or enforcement efforts, and related scientific findings were among frequently covered topics. Media narratives supporting smoke-free policies were frequently presented by messengers such as government, scientific, and civil society organizations and promoted their collaboration, such as in local expert trainings in Chinese cities. These actors often referenced public health interests, such as eliminating tobacco-caused death and disease, in calling for further enforcement efforts. Online news media articles often referred to populations vulnerable to SHS (e.g. mentioning protecting schools and children’s spaces) in framing smoke-free policy and SHS protection as a moral responsibility.

These findings suggest that media report implementation of smoke-free policies in LMICs mostly in a favorable light, while also discussing implementation challenges. Some articles described insufficient regulatory frameworks, government inaction, and cultural normalization of smoking, citing incidents of smoking violations in public spaces, lacking signage, and complaints from people who do not smoke but are affected by SHS. For instance, in Indonesia, failure of existing tools to monitor implementation data were cited, and in China, parents were found smoking near schools, suggesting normalization of tobacco use. These incidents suggest the critical importance of effective enforcement mechanisms as well as other strategies to enhance compliance.

In line with previous studies on the benefits of increased public engagement in enforcing smoke-free policies, we observed discussion of the potential efforts, such as local task forces and campaigns aimed at raising awareness, in supporting enforcement in LMICs^11-13^. Furthermore, new and cross-sector enforcement methods may address current issues in implementation. For instance, we observed coverage praising novel digital monitoring technologies, educational youth engagement campaigns, and community surveillance avenues in this sample. Future research should explore new strategies and their effectiveness in reducing SHS exposure, especially in LMICs, including various methods of utilizing media to promote tobacco control.

While a substantial number of city-level, regional wins were mentioned – such as city-level WHO World No Tobacco Day awards in China and governmental awards in Indonesia – articles praising a national law were rare. In some countries, articles discussed how despite lacking implementation, the public generally supported tobacco control policy, leading to good compliance, such as in Ukraine and Vietnam.

In addition to strengthening the implementation of smoke-free legislation, stronger policies are also needed to improve tobacco control in LMICs. One-fifth (21%) of articles in this study called for stricter policies, such as eliminating designated smoking areas and expanding bans to various tobacco and nicotine products, for instance, e-cigarettes and hookahs in Indonesia, Mexico, and Ukraine. These calls align with current literature demonstrating the need for stronger smoke-free policy. Stronger measures include country-specific smoke-free legislation due to varying levels of implementation and compliance and expansion of current policies to address use of electronic cigarettes, as well as other tobacco or nicotine products and industry tactics^26^.

Many online news media articles framed designated smoking areas (DSAs) as violations to the rights of people who do not smoke to be free from SHS, highlighting their ineffectiveness, lack of enforcement, and low compliance levels. Consistently, complete bans have been demonstrated to be easier to implement than partial bans that allow for DSAs^27^. Partial bans may fail to adequately protect individuals from SHS, pose organizational challenges, and allow for increased tobacco industry interference^26^. Media and other actors should advocate for similar nationallevel legislation requiring 100% smoke-free policies to fully protect people from a major cause of health burden.

Although media sentiments against smoke-free policies were less common in this sample, 7% of the articles discouraged smoke-free policy implementation, and 3% advocated for more lenient smoke-free policies. Some of these articles framed smoking and DSAs as essential to a fair market, but these claims are often unsupported by empirical data. Articles also captured tobacco industry efforts to mislead the public and downplay smoke-free policy effectiveness, suggesting online news can reflect harmful industry narratives in LMICs.

Media can be leveraged to amplify scientific evidence in countering narratives of economic loss surrounding smoke-free policies. Proactive dissemination and other strategic communication strategies have been shown to play a crucial role in sustaining policy momentum and discrediting industry-fueled economic arguments, shifting public opinion in favor of tobacco control legislation^19,25^.

### Limitations

The articles in this study cover a limited timeframe (March 2022 – September 2023) and only include 10 LMICs and do not fully capture long-term trends or broader regional variations. There may be other limitations associated with using the Tobacco Watcher tool for identifying articles, such as the scope of media sources included in its surveillance system. Only online news media were analyzed, excluding other sources such as print media, television, radio, and social media, which may also shape public discourse on smoke-free policies. Future research could expand the geographical scope and include a broader range of media formats and data sources such as social media and print sources for improved generalizability.

Furthermore, the online news media articles often provided surface-level coverage, lacking depth or detailed analysis of policy implementation challenges and effectiveness. Variability in the quality and detail of articles can affect the consistency of the media narratives analyzed. In addition, articles published in languages other than English were translated using Google Translate, which may have resulted in uncaptured nuanced meanings in policy discussions.

Ultimately, news media can be utilized as avenues for building public support for policy implementation and new policies and countering tobacco industry narratives. Governments, health organizations, and other public health advocates should leverage media to accessibly communicate tobacco control progress and obstacles.

## CONCLUSIONS

This study provides insight into the landscape of online news media articles on smoke-free policy implementation in LMICs in 2022–2023, with most supporting 100% smoke-free public places. While online media reported implementation barriers such as lacking enforcement, insufficient policy, and cultural normalization of smoking, they also highlighted positive impacts of smoke-free law and progress. Smoke-free laws have demonstrated the impacts of encouraging smoking cessation, denormalizing tobacco use, decreasing health risks, and improving economic revenue for businesses and tourism. These outcomes were captured by news narratives that emphasized broader public health and tobacco control goals. Strategic use of news media may serve to amplify evidence and support for such smoke-free policies in LMICs and critically urge governments to pass tobacco control legislation that can save lives.

## Supplementary Material



## Data Availability

The data supporting this research are available from the following sources: https://tobaccowatcher.globaltobaccocontrol.org/
