# Supplementary material for: Content analysis of media coverage on smoke-free policy implementation in ten low- and middle-income countries
Source: Tob Induc Dis. 2025 Nov 25;23:10.18332/tid/211700. doi: 10.18332/tid/211700 (PMC12648208; doi:10.18332/tid/211700)
Supplement: Supplementary file 1 [file TID-23-183-s1.pdf]

**Codebook with code definition**

| Category | Variable | Definition |
|---|---|---|
| **Relevancy** | Relevancy | Article either features multiple paragraphs discussing smoke-free policy implementation in general or takes a clear position on smoke-free policy implementation (e.g. mentioning a specific policy and claiming it is working well to fight death/disease). |
| **Type of article** | News | Article is a news report written to inform readers regarding recent events; usually written in a neutral tone. |
| | Feature | Article is a news report that dives more in-depth into an event or issue compared to news reports, oftentimes contains bias/a point of view, is less formal, and more creative. |
| | Opinion | Article is a piece written to convey a specific person's insights or point of view on a specific topic, is usually informal and written with personality, such as editorials. |
| | Other | End coding if it's a research journal article<br>Article is not a news/feature/opinion article. |
| **Argument position** | Supports implementation of existing smoke-free policy | Statements that advocate for or promote the continued or improved enforcement of current smoke-free policies. This includes explicit endorsements, recommendations for stricter enforcement, or any expression of encouragement for the ongoing application of the existing smoke-free regulations. Article supports stronger enforcement of the current (existing) smoke-free policies as they are, rather than suggesting new policies or changes. |
| | Discourages implementation of existing smoke-free policy | Statements that reject current/existing smoke-free policy measures in general (might be rare in our sample). This code is used for things like, "tobacco control measures hurt the economy so should not be implemented." |
| | Calls for change – stricter smoke-free policy | Statements that suggest the need for stronger or stricter smoke-free policies to strengthen tobacco control. Suggests a change or new proposal from current policy. |

| | | |
|---|---|---|
| | Calls for change – less smoke-free policy | Statements that suggest altering current smoke-free policies by proposing a relaxation or reduction in their stringency (might be rare in our sample). Calls for a change from current policy that results in less strict tobacco control measures. |
| | Successful implementation of smoke-free policy | Statements that existing smoke-free policy implementation is being carried out well; this could be implied through earning a recognition or award for the achievements or efforts in implementing smoke-free policies. Note that it is possible for a positive evaluation of current efforts to be presented alongside further changes needed. |
| | Unsuccessful implementation efforts for smoke-free policy | Statements that existing smoke-free policy implementation is not being carried out well. |
| **Argument presenters** | Government | Article directly or indirectly refers to statements made by the government in association with smoke-free policy implementation. This includes government agencies at local, regional, state, or country levels. |
| | Tobacco industry | Article directly or indirectly refers to statements made by the tobacco industry in association with smoke-free policy implementation. |
| | Doctors/hospitals | Article directly or indirectly refers to statements made by healthcare providers, including physicians or other healthcare workers and hospitals or other healthcare facilities, in association with smoke-free policy implementation. |
| | People who consume tobacco | Article directly or indirectly refers to statements made by people who consume tobacco in association with smoke-free policy implementation. |
| | People who don't smoke but are affected by others' smoking | Article directly or indirectly refers to statements made by people who don't smoke but are affected by others' smoking such as mentions of second-hand smoke in association with smoke-free policy implementation. |

| | | |
|---|---|---|
| | Researchers/experts | Article directly or indirectly refers to statements made by tobacco control research experts in association with smoke-free policy implementation. Include professors, universities, research institutes, PhDs; exclude clinicians or healthcare workers. |
| | Research/studies | Article directly or indirectly refers to statements made by a specific research study or research studies in general in association with smoke-free policy implementation. |
| | Civil society organizations | Article directly or indirectly refers to statements made by organizations that's not a government agency, including tobacco-control organizations such as Campaign for Tobacco-Free Kids, advocacy associations (tobacco-focused or not), or other health organizations in association with smoke-free policy implementation. This includes non-profit organizations, NGOs (non-governmental organizations), foundations, and any other group that's non-government. Exclude research institutes. |
| | World Health Organization | Article directly or indirectly refers to statements made by the World Health Organization or WHO sub-offices in association with smoke-free policy implementation. |
| | Other | Article directly or indirectly refers to statements made by other persons in association with smoke-free policy implementation (e.g. family members of people who consume tobacco). For instance, lawyers, family members of people who consume tobacco, etc. |

**© 2025 Xing E. et al.**
